# Miniscrew-assisted rapid palatal expansion (MARPE): how to achieve greater stability. In vitro study

**DOI:** 10.1590/2177-6709.26.1.e211967.oar

**Published:** 2021-03-22

**Authors:** Flávio de Mendonça COPELLO, Daniel Paludo BRUNETTO, Carlos Nelson ELIAS, Matheus Melo PITHON, Raildo Silva COQUEIRO, Amanda Cunha Regal de CASTRO, Eduardo Franzotti SANT’ANNA

**Affiliations:** 1 Universidade Federal do Rio de Janeiro, Departamento de Odontopediatria e Ortodontia (Rio de Janeiro/RJ, Brazil).; 2 Universidade Federal do Paraná, Departamento de Ortodontia (Curitiba/PR, Brasil).; 3 Instituto Militar de Engenharia (Rio de Janeiro/PR, Brazil).; 4 Universidade Estadual do Sudoeste da Bahia, Departamento de Ortodontia (Jequié/BA, Brazil).

**Keywords:** Dental materials, Orthodontic anchorage procedures, Palatal expansion technique

## Abstract

**Objective:**

Assess the influence of mono- and bicortical anchorage and diameter of mini-implants (MIs) on the primary stability of these devices.

**Methods:**

60 self-drilling MIs were distributed in six groups according to diameter (1.5mm, 1.8mm or 2.0mm) and type of anchorage (monocortical and bicortical) in bovine rib. The primary stability was evaluated by insertion torque, micromobility and pull-out strength tests. ANOVA and/or Tukey analysis were used to conduct intergroup comparisons (*p*< 0.05). Non-parametric statistics (Kruskal-Wallis and Mann-Whitney) were performed when normality was not found (*p*< 0.05).

**Results:**

MIs with larger diameters and bicortical anchorage showed greater primary stability regarding insertion torque (*p*< 0.05) and micromobility (*p*< 0.05). Only MI diameter had an effect on the pull-out strength test. Larger diameter MIs presented better retention in pull-out strength tests (*p*< 0.001), regardless of mono- or bicortical anchorage.

**Conclusions:**

MI primary stability is dependent on its diameter and type of anchorage. Bicortical anchorage showed greater stability when compared with monocortical anchorage, independently of other variables.

## RESUMO

**Objetivo:**

Avaliar a influência da ancoragem mono e bicortical e do diâmetro dos mini-implantes (MIs) na estabilidade primária desses dispositivos.

**Métodos:**

60 MIs autoperfurantes foram distribuídos em seis grupos, de acordo com o diâmetro (1,5 mm, 1,8 mm ou 2,0 mm) e tipo de ancoragem (monocortical e bicortical), e inseridos em costela bovina. A estabilidade primária foi avaliada pelos testes de torque de inserção, micromobilidade e resistência à tração. ANOVA e/ou análise de Tukey foram usadas para realizar comparações intergrupos (*p*< 0,05). Estatística não paramétrica (Kruskal-Wallis e Mann-Whitney) foi realizada quando a normalidade não foi encontrada (*p*< 0,05).

**Resultados:**

MIs com diâmetros maiores e ancorados bicorticalmente apresentaram maior estabilidade primária em relação ao torque de inserção (*p*< 0,05) e micromobilidade (*p*< 0,05). Apenas o diâmetro do MI influenciou os resultados do teste de resistência à tração. MIs de maior diâmetro apresentaram melhor retenção nos testes de resistência à tração (*p*< 0,001), independentemente da ancoragem mono ou bicortical.

**Conclusões:**

a estabilidade primária do MI é dependente de seu diâmetro e tipo de ancoragem. A ancoragem bicortical apresentou maior estabilidade quando comparada à ancoragem monocortical, independentemente das demais variáveis.

## INTRODUCTION

Orthodontic mini-implants (MIs) have greatly impacted orthodontic biomechanics and anchorage, since their advent. Movements that were very limited before, such as molar intrusion, became possible, and other routinely performed movements, such as molar distalization, were optimized.^1^

It is known that 20% of mixed dentition patients have maxillary constriction,^2^ and the most popular treatment is rapid maxillary expansion (RPE). When RPE with a tooth-borne appliance is used to treat adolescents and young adults, it produces 35% skeletal orthopedic expansion and 65% dentoalveolar tipping.^3^ RPE skeletal effects diminish with patient aging, because of the progressive calcification and interdigitation of circummaxillary sutures, and the decreased elasticity of bone in adults.^4^

In adult patients, where there is no potential for mid-palatal suture opening using conventional techniques, the treatment option is surgically-assisted rapid palatal expansion (SARPE).^5^ However, this is a more invasive technique with considerable side effects, such as injury to the periodontium, root resorption,^6^ sinus infection,^7^ and injury to the branches of the maxillary nerve.^8^ In addition, relapse of the transverse maxillary dimension has been demonstrated in the short term.^8^ In 2010, MIs were associated with rapid palatal expanders for the first time^9^ and are still yielding promising results. This expansion technique, known as miniscrew-assisted rapid palatal expansion (MARPE), can make the expansion more efficient in adolescents and young adults, and more feasible in elderly adults.^10^ When well indicated, this technique can become a potential alternative to SARPE.^9^ ^,^ ^11^

From a clinical point of view, bicortical anchorage should be used in cases where heavy anchorage is desired.^12^ The use of MIs allows tooth-bone-borne palatal expanders to apply forces directly into the basal bone, thus bringing horizontal expansion forces close to the midpalatal suture and right into the maxillary center of resistance.^9^ Thus, MI stability is essential to resist the magnitude of the applied mechanical forces required to open the heavily interdigitated circummaxillary sutures. 

However, with the promising use of MARPE on the rise, many doubts regarding technical specifications have arisen, such as: What is the most appropriate length and diameter of the MI?; How deep should the MI be inserted into the bone?; What is the best mechanical position for the jackscrew in the sagittal and vertical planes? These questions should be addressed scientifically by laboratorial and clinical trials.

Few laboratorial studies have demonstrated that the MI diameter has a direct influence on its primary stability, and others have suggested that bicortical anchorage might impact it as well.^13^ ^,^ ^14^ However, to our knowledge, no study has assessed the influence of these two factors simultaneously on MI primary stability. Our hypothesis is based on the possibility that larger diameters MI could positively influence the stability of these devices, as well as the bicortical anchorage.

The aim of this study was to compare the effects of monocortical and bicortical anchorage of MIs with different diameters on their primary stability, through mechanical *in vitro* tests.

## MATERIAL AND METHODS

The project was approved by the Animal Ethics Committee of the Center for Health Sciences of the Federal University of Rio de Janeiro before the study began, under number 01200.001588/2013-87.

**Figure 1:**
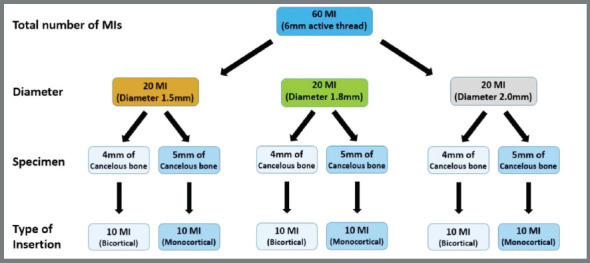
Flowchart showing distribution of specimens into the groups/subgroups according to their diameter and type of insertion.

**Figure 2:**
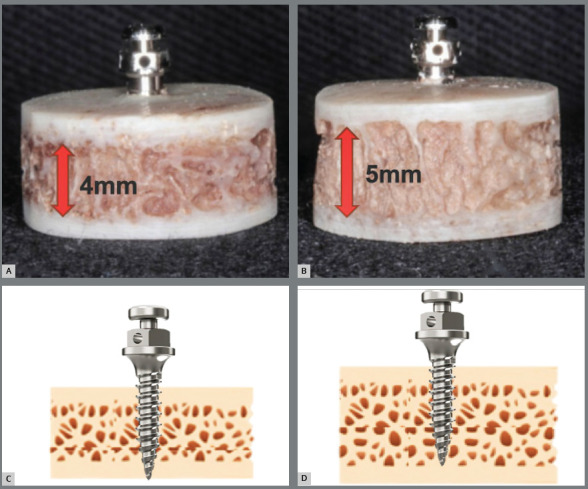
Specimens simulating palate according to trabecular bone thickness and mini-implant insertion depth diagram. A) 4 mm B) 5 mm C) Bicortical anchorage D) Monocortical anchorage.

Sixty commercially available cylindrical self-drilling MIs (6 mm length) made of Ti-6Al-4V alloy (Conexão Implantes, São Paulo, SP, Brazil), were allocated into six groups (n=10), according to their diameter and insertion depth (monocortical or bicortical) (Fig 1). The number of samples was calculated using the sample size data of a previous pilot study (SD = 0.06, α = 5%, power of study = 80%). Sixty sections (8 mm ø) were removed from a bovine rib (*Bos taurus indicus*, Nelore lineage) with a trephine bur (8 mm ø x 20 mm long, Sin Implantes, São Paulo, SP, Brazil) and stored by freezing (-20ºC) (Fig 1). All the specimens had approximately 1 mm of cortical bone (on the top and bottom) and two types of trabecular bone length (4 mm and 5 mm) to achieve the monocortical or bicortical insertion procedure (Fig 2).

### ASSESSMENT OF MINI-IMPLANT PRIMARY STABILITY

#### Insertion torque (IT)

The MI sites were predrilled with a lance (Orthodontic Kit, INP system, São Paulo, Brazil) to a depth of 1 mm, following the protocol of a previous study.^15^ The insertion was conducted by a single operator by using a manual key connected to a digital torque meter (Lutron TQ-8800, Taipei, Taiwan). Each MI was inserted until all the threads were fully contained in the block. A mechanical device was used to align the torque meter, the MI and the bone blocks, maintaining the system in a perpendicular relationship. The peak insertion torque values were recorded in Newton centimeters (Ncm). 

#### Mini-implant mobility

MI mobility was evaluated with the Periotest^®^ instrument (Medizintechnik Gulden, Modautal, Germany). A special acrylic device was used to fix both the sample and Periotest^®^ handpiece, and to standardize the distance between the sleeve and the MI.^16^ The handpiece was calibrated before each screw was measured. Two recordings were collected for each MI, and the average value was designated as the Periotest value (PTV), ranging on a scale from -8 to +50, where the smaller the PTV value, the smaller the micromobility and the higher the primary stability.

#### Pull-out strength (PS)

This test was conducted in a universal testing machine (EMIC DL 2000, São José dos Pinhais, PR, Brazil) connected to a 500 Kgf load cell. Two stainless steel devices were developed especially for the purpose of maintaining exact axial coincidence of the system. A crosshead speed of 5 mm per minute was selected, based on the American Standard Specification and Test Methods (F543-07) guidelines for metallic medical bone screws, and the maximum PS was recorded in Newtons (N).

### STATISTICAL ANALYSIS

Statistical analysis was performed with the SPSS software (version 22, SPSS Inc, Chicago, IL, USA). The two-way ANOVA test was used to evaluate the interaction of the MI diameter (1.5 mm, 1.8 mm and 2.0 mm) and the insertion depth (monocortical or bicortical). Normality was verified by the Kolmogorov-Smirnov test, and homogeneity of variances, by the Levene test. When the main effect was observed for the diameter factor (no interaction), the *post-hoc* Tukey test was used to determine intergroup comparisons. When an interaction was verified between diameter and bone insertion, the effect of the interaction was contrasted to determine the differences between the groups. When normality and/or homogeneity of variances was violated, a nonparametric statistic was applied: Kruskal-Wallis test with the comparisons between pairs analyzed by the Mann-Whitney test. The level of significance was set at 5%. 

## RESULTS

**Figure 3:**
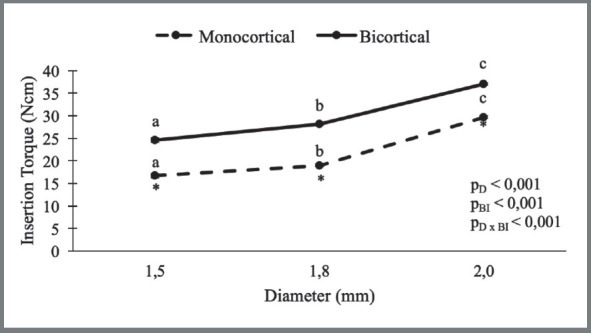
Graph showing insertion torque ( IT ) analysis (D=Diameter; BI=Bone Insertion). Higher IT values were found in mini-implants with greater diameter and bicortical insertion. * ANOVA two-way: a, b, c distinct letters indicate statistical difference (p ≤ 0.05).

The insertion torque results are displayed in Figure 3. Mechanical performance was clearly influenced by MI diameter and type of anchorage, given that higher insertion torque values were found in devices with greater diameter and bicortical insertion (*p*< 0.001). In addition, the insertion torque values for all the diameters evaluated were higher in the MIs with bicortical insertion (Bicortical: 1.5ø: 24.61 ± 0.47; 1.8ø: 28.13 ± 0.18; 2.0ø: 37.00 ± 0.19 / Monocortical: 1.5ø: 16.66 ± 0.45; 1.8ø: 18.95 ± 0.33; 2.0ø: 29.67 ± 0.34).

**Figure 4:**
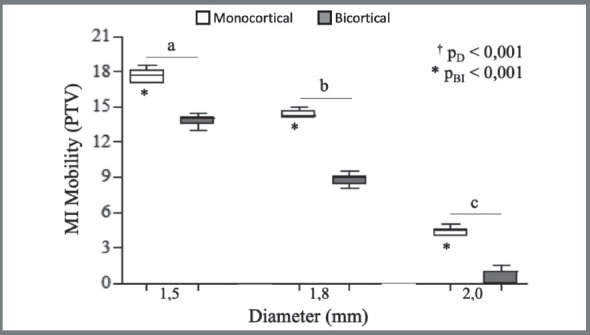
Graph showing Periotest assessment (D= Diameter; BI=Bone Insertion). The results indicated influence of diameter and insertion type on mobility. Mini-implant mobility was statistically lower for mini-implants with larger diameters regardless of anchorage insertion type. * Kruskal-Wallis test: a, b, c distinct letters indicate statistical difference between the diameters (Mann-Whitney test); ^†^ Mann-Whitney test.

Mobility (Fig 4) was influenced by insertion type and MI diameter. MI mobility for both types of insertion decreased as diameter increased. The lowest mobility was found in the 2.0-mm diameter MI with bicortical insertion (Bicortical: 1.5ø: 14.00 ± 0.06; 1.8ø: 9.00 ± 0.80; 2.0ø: 1.00 ± 1.00 / Monocortical: 1.5ø: 17.75 ± 1.10; 1.8ø: 14.25 ± 0.60; 2.0ø: 4.50 ± 0.60).

**Figure 5:**
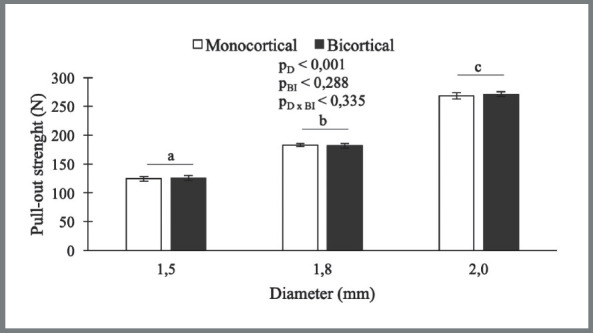
Graph showing pull-out strength results. (D= Diameter; BI=Bone Insertion). Only mini-implant diameter influenced pull-out strength values.

Only MI diameter influenced pull-out strength values; MIs with larger diameter were more resistant to traction (Fig 5), regardless of mono- or bicortical insertion. (Bicortical: 1.5ø: 125.58 ± 4.84; 1.8ø: 181.87 ± 3.98; 2.0ø: 271.41 ± 3.70 / Monocortical: 1.5ø: 124.23 ± 4.10; 1.8ø: 182.78 ± 2.87; 2.0ø: 268.40 ± 5.05).

**Table 1:** Descriptive statistics for Insertion Torque and Pull-out Strength.

Primary Stability Tests	BI	Diameter (D)			Effect	*p-valor
		1.5 mm	1.8 mm	2.0 mm		
Insertion Torque (Ncm)					D	< 0.001
	MC	16.66 ± 0.45^a^	18.95 ± 0.33^b^	29.67 ± 0.34^c^	BI	< 0.001
	BC	24.61 ± 0.47^a†^	28.13 ± 0.18^b†^	37.00 ± 0.19^c†^	D x BI	< 0.001
Pull-out Strength (N)		a	b	c	D	< 0.001
	MC	124.23 ± 4.10	182.78 ± 2.87	268.40 ± 5.05	BI	0.288
	BC	125.58 ± 4.84	181.87 ± 3.98	271.41 ± 3.70	D x BI	0.335

D, Diameter; BI, Bone Insertion; D x BI, effect between diameter and bone insertion; MC, monocortical; BC, bicortical. Results are expressed as mean ± standard deviation. * ANOVA two-way: a, b, c distinct letters indicate statistical difference (p ≤ 0.05) between the diameters, according to the comparisons for interaction effect (insertion torque) and Tukey test (pull-out strength) ; ^†^ indicates statistical difference (p ≤ 0.05) between types of bone insertion.

**Table 2:** Descriptive statistics for mini-implant micromobility.

Bone Insertion	Diameter			*p-valor
	1.5 mm	1.8 mm	2.0 mm	
Monocortical	17.75 ± 1,10^a^	14.25 ± 0,60^b^	4.50 ± 0.60^c^	< 0.001
Bicortical	14.00 ± 0,06^a^	9.00 ± 0,80^b^	1.00 ± 1.00^c^	< 0.001
^†^p-valor	< 0.001	< 0.001	< 0.001	

The results are expressed as median ± interquartile amplitude. * Kruskal-Wallis test: a, b, c distinct letters indicate statistical difference between the diameters (Mann-Whitney test); ^†^ Mann-Whitney test.

## DISCUSSION

With the introduction of the MARPE technique as a possible alternative to SARPE, several studies have been published to evaluate its efficacy in treating transverse maxillary deficiency.^10^ ^,^ ^17^ ^-^ ^20^ Adequate MI stability is imperative for resisting the loads employed during activation of the expander, especially in adults, where greater interdigitation of the sutures requires higher mechanical loads.

In the present study, the MIs were selected with the same length of active threads to standardize both the insertion, with all the active threads of the MI inserted into the bone, and the same transmucosal portion leading out of the specimen, in order to reduce the moment of force variable.

We used bovine rib because it has been validated as a bone model in other biomechanical studies.^21,22^ In addition, the thickness of the bovine rib in selected areas allows the simulation of monocortical and bicortical anchorage. 

Since the MARPE technique is relatively recent, the primary stability and mechanical performance of the MI must be evaluated when it is correlated with the type of anchorage (mono- and bicortical). In this study, primary stability parameters such as insertion torque, Periotest^®^ and pull-out values were used as stability predictors.^23^ ^,^ ^24^

Studies with finite element methods (FEM) were used to simulate the effectiveness of the midpalatal opening, the expansion resistance and the MI stability when using a tooth-bone-borne palatal expander.^12^ ^,^ ^25^ ^,^ ^26^ The present study corroborates previous reports that used FEM^12,25^ with better mechanical results (insertion torque and Periotest^®^ values) for MIs inserted with bicortical versus monocortical anchorage. The study by Lee et al.^12^ showed that bicortical anchorage was more effective to open the midpalatal suture and to prevent the distortion of the device, in comparison with monocortical anchorage. Therefore, a positive correlation seems to exist between MI stability and effectiveness of the expansion. Specifically, greater MI stability provides better device resistance against expansion forces. Furthermore, the positive correlation observed between bicortical anchorage and low Periotest^®^ values (p < 0.05) indicates the potential of bicortical anchorage against lateral forces.

In contrast, a study by Poorsattar-Bejeh^26^ using FEM showed that monocortical anchorage provided greater stability compared with bicortical anchorage, based on the pull-out test during FEM simulation. In our study, although the results showed better mechanical performance of MIs when inserted with bicortical anchorage, differences were not statistically significant. 

A positive correlation was found in different studies between larger MI diameter and better mechanical performance.^27^ ^-^ ^29^ Pimentel et al.^14^ found (*in vitro*) that all MIs with a diameter of 1.8 mm, 2.0 mm and 2.2 mm, used with the MARPE technique, endured loads beyond those clinically necessary for breaking loose the midpalatal suture in maxillary expansion. We found that MIs with a diameter of 2.0 mm and 1.8 mm inserted with monocortical anchorage had better mechanical outcomes, compared with a 1.5 mm diameter inserted with bicortical anchorage. Therefore, MIs with larger diameters are recommended, since they deliver good mechanical stability, and since bicortical anchorage may not always be clinically achieved due to the sensitivity of the technique. Moreover, MIs of greater diameter should be used with tooth-bone-borne expanders in the anterior portion of the palate, where bicortical anchorage is not always possible. 

It is known that the results achieved with primary stability influence secondary stability and permanence of the MI. When the MI is well stabilized to allow it to receive the necessary load, the chances of successful treatment are greater.^30^

Because of the inherent limitations of *in vitro* studies and mechanical tests, future studies using conventional clinical model analysis are needed to confirm our results. We also suggest that a mechanical *in vitro* analysis of the MI be conducted using the MARPE expander, bearing in mind that a microstructural assessment of the bone should also be made when this type of device is used. 

## CONCLUSIONS

The following conclusions can be drawn:

- Mini-implant primary stability is dependent on the diameter and the type of anchorage (mono- or bicortical) of the device.
- Mini-implants inserted with bicortical anchorage had better mechanical results, compared with monocortical anchorage
- Devices with a 2.0-mm diameter had better results, even when bicortical anchorage was not achieved.
